# A Prospective study of the National Early Warning Score in Cardiothoracic Surgery

**DOI:** 10.1186/1749-8090-10-S1-A344

**Published:** 2015-12-16

**Authors:** M Tarazi, N Chan, N Mayooran, R Neagu, B Philip, MN Anjum, K Doddakula

**Affiliations:** 1 Department of Cardiothoracic Surgery, Cork University Hospital, Cork, Republic of Ireland

## Background/Introduction

The National Early Warning Score (NEWS) is a clinical guide used to facilitate early detection of deterioration by categorising a patient's severity of illness and prompting nursing staff to request a medical review at specific trigger points utilising a structured communication tool while following a definitive escalation plan. Adopting a National Early Warning Score (NEWS) is beneficial for standardising the assessment of acute illness severity, enabling a more timely response using a common language across acute hospitals nationally.

## Aims/Objectives

To determine whether the NEWS guideline recommendations apply to post-operative cardiac and thoracic patients.

## Method

A prospective study of patients undergoing cardiac and thoracic surgery was performed. Data was entered into a spread sheet using patient's medical notes and observation sheets. The data looked at procedure performed, post-operative length of stay in ICU, post-operative complications, NEWS score on arrival to ward, and average NEWS score each day up to and including day of discharge.

## Results

100 post-operative cardiac patients and 100 post-operative thoracic patients were included in the study. Our results showed initially post-operative cardiac and thoracic patients had a high NEWS score that resolved during their hospital stay with no intervention or urgent medical review required.

## Discussion/Conclusion

Our results show that the NEWS score guide does not correlate with cardiac and thoracic patients in the initial post-operative period. More research in this area could shed some light on potential adjustments and modifications to correlate better with our patient population.

